# Obesity does not influence prostate intrafractional motion

**DOI:** 10.1002/jmrs.255

**Published:** 2018-01-23

**Authors:** Amy Brown, Alex Tan, Scott Cooper, Andrew Fielding

**Affiliations:** ^1^ Townsville Cancer Centre The Townsville Hospital Townsville Queensland Australia; ^2^ James Cook University Townsville Queensland Australia; ^3^ Queensland University of Technology Brisbane Queensland Australia

**Keywords:** Interfraction motion, intrafraction motion, obesity, prostate cancer, radiotherapy

## Abstract

**Introduction:**

Motion of the prostate is problematic in the accurate delivery of external beam radiation therapy (EBRT) for prostate cancer. This study investigated the relationship between body mass index (BMI), an easily measured indicator of obesity, and prostate motion.

**Methods:**

Prostate motion during EBRT was assessed by measuring the displacement of fiducial markers implanted within the prostate in 130 prostate cancer patients. Interfractional motion was corrected on daily imaging through pre‐treatment cone‐beam‐computed tomography (CBCT) and intrafractional motion measured using movie sequences captured using an electronic portal imaging device (EPID) during treatment delivery.

**Results:**

There was no statistically significant relationship between the mean intrafractional motion and BMI, except in the left‐right (LR) translation (*P* = 0.049) over the study population. For each BMI category, there was no statistical significance (*P* > 0.05) between any of the translations/rotations except LR (*P* = 0.003).

**Conclusion:**

While intrafractional motion is an important consideration, prostate motion cannot be reliably predicted through measurement of patient's BMI.

## Background

External beam radiation therapy (EBRT) is one of the primary treatments options of prostate cancer.^1^ The prostate is known to move within the pelvis due to factors such as bladder and bowel filling. As such, daily image‐guided radiation therapy (IGRT) is recommended.^1^, ^2^

Obesity is an increasing global epidemic, with implications in the treatment of prostate cancer. Obese men have significantly greater chance of dying of prostate cancer than non‐obese men,^3^ and are approximately twice as likely to develop a high‐grade prostate cancer.^4^ Obesity is associated with a 98% increase in prostate cancer risk, after adjusting for lower prostate‐specific antigen (PSA) and a larger prostate size.^5^ In addition, overweight or obese prostate cancer patients are more likely to be younger and have co‐morbidities such as hypertension and diabetes.^6^

Obesity limits the treatment options available to the prostate cancer patient, with a higher Body Mass Index (BMI) increasing the operative risk associated with a radical prostatectomy.^7^ The most widely used non‐surgical treatment is radiation therapy. There is, however, significantly greater set up variability in obese patients compared to less obese patients when treating with radiation therapy due largely to the variability in location of external pelvic skin markers (used to set up the patient on a daily basis) relative to internal anatomy.^8^ Overweight and obese patient groups show a significant difference in interfractional prostate shift on a daily basis.^9^

This study aimed to investigate the relationship between obesity as measured by BMI and intrafractional prostate displacement over the course of a radiation therapy treatment.

## Methods

The study was reviewed and approved by the institute's Human Research and Ethics Committee. A total of 130 patients of differing BMI were recruited and provided written consent over a 3‐year period (2011–2013). Of the 176 prostate patients screened, 22 patients did not meet eligibility criteria and 24 patients declined participation. BMI was calculated using the World Health Organisation (WHO) definition of the weight (kilograms) divided by the square of the height (metres).^10^ Patients were then categorised in the database according to the four major WHO classifications (underweight, normal, overweight and obese). Patients were excluded from the study if they had previous or concurrent cancers within the pelvis, hip prosthesis, ECOG performance status greater than 2, or if dose constraints to critical structures were unachievable. Dose constraints had to be met for inclusion in this study so as not to negatively impact the clinician‐graded side effects and patient‐reported quality of life, a secondary analysis of this study not reported in this paper.

Patients had three gold seed fiducial markers (*CIVCO*, Iowa, USA), 1 × 3 mm in size, inserted into the prostate for IGRT. Patients were simulated according to departmental protocol, positioned supine on the solid carbon‐fibre CT couch top, with a standard head rest, arms on chest, kneefix under knees and footstocks supporting feet and ankles. The standard departmental bowel and bladder preparation protocol was adhered to, including an empty rectum/bowel (achieved by taking Movicol laxative everyday throughout treatment) and full bladder (achieved by emptying their bladder then drinking 300 mL of water 20 min prior to their appointment).

Patients were planned on the XiO planning system (*Elekta CMS*, Missouri, USA) according to departmental protocols. A 3D conformal technique was used, with two laterals (or posterior obliques), two anterior obliques and a direct anterior beam. Treatment was delivered on the department's megavoltage (10 MV) linear accelerators. Patients were queried each day as to their adherence to the bladder and bowel protocol. If the patient reported troubles, particularly in emptying their bowel that day, alterations to the aperient schedule were considered. Treatment times varied throughout the course of treatment, between the hours of 8 am and 8 pm.

### Imaging methods

The correction of interfractional prostate displacement was performed by daily Cone Beam CT (CBCT) verification imaging, matching to the fiducial markers. Bladder and bowel volumes were assessed daily on the CBCT, and corrective action taken by treatment staff if this varied significantly from planned volume. Based upon the CBCT match, the patient set up was corrected by shifting the couch remotely. Movements were performed on any parameter greater than 1 mm. The treatment couch could only correct for the three translational displacements and not rotational displacements, and so a ‘best‐fit’ match was performed by the treating radiation therapists where rotation was observed. If the CBCT functionality was not operational on a certain fraction, the patient's set up was verified and corrected utilising kV or MV matches, requiring a manual adjustment of the couch with an action level of 3 mm. These fractions were excluded from the analysis. At the discretion of treatment staff, if significant difference between bladder or rectum volume was observed, the patient was taken off the treatment couch for further remedial action prior to treatment delivery. This was at the discretion of the treatment staff, with no set protocols to measure significant differences.

Intrafractional motion was measured using movie loops captured during the delivery of the anterior and lateral (or posterior oblique) treatment fields using the iView electronic portal imaging device (EPID) (Elekta, Stockholm, Sweden). Images were acquired approximately once every 5–10 sec during treatment delivery, allowing for the measurement of any prostate movement during this time. An average of 7–8 frames were acquired for each lateral field, and 2–3 for the anterior field, however, this was dependent upon each individual's plan and treatment delivery.

**Figure 1:**
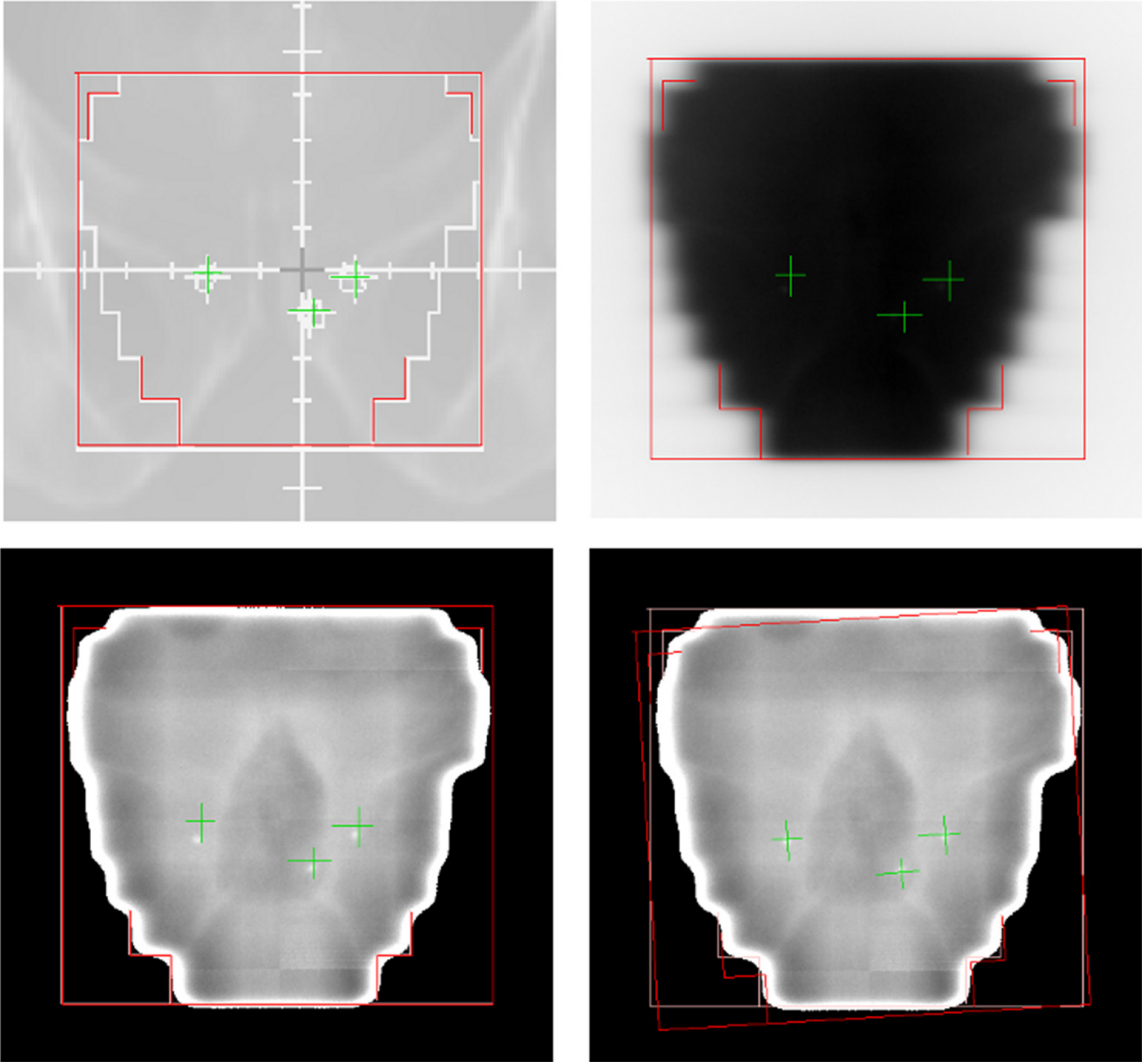
Example of rotation measure in iView.

The principal investigator alone measured the intrafractional motion on the movie captures to eliminate inter‐user variability. Intra‐user variability was analysed by measuring the intrafraction motion on eight sets of images of four different patients (2 different images per patient). The investigator was blinded to the patient's BMI during the measurement process to limit any bias in measuring prostate motion. As the movie captures were of the treatment fields, there could be some subjective image quality differences noted between patients of different sizes, however, the patient's BMI was not discernible from the quality of the movie capture image. Where prostate rotation and/or deformation were visible, the best fit was measured on the movie captures. This was achieved aligning the FMs using the rotation feature in iView (Fig. 1). Due to the planes analysed, pitch and yaw could be measured, however, roll could not be measured. As there was no rotational correction applied at CBCT, the CBCT measured rotation was subtracted from the movie capture rotation measurement, to indicate the true intrafraction rotation. A correction for gantry sag at the lateral and posterior oblique movie capture angles was applied, whereby the measured gantry sag (0.75–1.25 mm) at the appropriate angle was subtracted from the measured data.

### Statistical methods

Sample size was calculated to detect a displacement difference of greater than 5 mm, for a level of significance of 0.05 and power of 80%. The overall population mean and the mean intrafractional displacements of each of the four BMI patient groups were assessed for statistically significant difference utilising linear regression analysis and analysis of variance (ANOVA). While ANOVA determined if the BMI groups were different, Tukey post hoc tests were then performed to determine which groups were different, as the assumption of homogeneity of variances was not violated. Frequency and range data within 3 mm, 5 mm and 10 mm tolerances were also assessed. All data were collected and analysed in SPSS Version 19.0 software (IBM, Armonk, NY). Where there were missing data for any of the components (CBCT or Movie captures), the partial data were utilised for analysis.

## Results

### Demographics and data

The mean BMI was 29.4 kg/m^2^ across the study cohort (range of 18.22–47.00 kg/m^2^). As there was only one underweight patient, the underweight and normal BMI categories were combined for analysis. A total of 16 patients (12.3%) had less than three fiducial markers. Further patient demographics are found in Table 1. Across all patients, a total of 4357 out of 5038 fractions (86.5%) had complete or partial movie capture data collected. The number of fractions with missing movie capture data across BMI categories are tabulated in Table 1. Intra‐user variability was measured at 0.44 mm, 0.38 mm and 0.73 degrees in the horizontal, vertical and rotational planes respectively.

**Table 1:** Patient characteristics

Parameter	Group	Total population (Percentage)	Underweight/Normal	Overweight	Obese
BMI Classification		130	*Underweight*: 1 (0.8%) *Normal:* 24 (18.5%)	56 (43.1%)	49 (37.7%)
Dose (Gy)	78	109 (83.8%)	17	45	47
	76	10 (7.7%)	2	7	1
	74	11 (8.5%)	6	4	1
Seminal Vesicles Inclusion	Entire course	34 (26.0%)	7	13	14
	Partial course	73 (56.0%)	11	33	29
	Not included	23 (18.0%)	6	11	6
Fiducial Markers	3 markers	114 (87.7%)	23	51	40
	2 markers	14 (10.8%)	3	4	7
	1 marker	2 (1.5%)	0	1	1
Number of Fractions with missing movie capture data		681 (13.5%)	213 (4.2%)	232 (4.6%)	237 (4.7%)

The mean and standard deviation for the overall population in each intrafractional translation and rotation is detailed in Table 2. Patient BMI could not significantly predict mean intrafractional motion except for in left‐right (LR) translation (*P* = 0.049) through linear regression analysis, as summarised in Table 3.

**Table 2:** Intrafractional mean and standard deviation across overall study population and by BMI category

	Overall population Mean ± SD	Underweight & Normal Mean ± SD	Overweight Mean ± SD	Obese Mean ± SD
LR (mm)	0.37 ± 0.83	0.35 ± 0.79	0.11 ± 0.76	0.68 ± 0.86
AP (mm)	0.34 ± 1.48	0.31 ± 1.51	0.19 ± 1.59	0.49 ± 1.38
SI (mm)	−0.90 ± 1.41	−1.34 ± 2.10	−0.73 ± 1.08	−1.02 ± 1.12
Pitch (deg)	−0.84 ± 3.57	−1.55 ± 3.83	−1.16 ± 3.97	−0.08 ± 2.83
Yaw (deg)	−0.40 ± 2.00	−0.04 ± 1.67	−0.54 ± 1.93	−0.42 ± 2.25

LR, left‐right; AP, anterior‐posterior; SI, superior‐inferior.

**Table 3:** Results for mean intrafractional linear regression analysis

	*R* square	Adjusted *R* square	Slope coefficient	Confidence interval (95%)	Statistical significance *F*(1,130)
LR (mm)	0.032	0.024	0.029	0.0 to 0.057	3.945, *P =* **0.049**
AP (mm)	0.033	0.025	0.049	0.001 to 0.098	4.155, *P =* 0.440
SI (mm)	0.000	−0.008	0.007	−0.038 to 0.052	0.050, *P =* 0.823
Pitch (deg)	0.17	0.009	0.087	−0.035 to 0.21	1.992, *P =* 0.161
Yaw (deg)	0.000	−0.009	0.003	−0.069 to 0.076	0.009, *P =* 0.926

The bold indicates a significant result. LR, left‐right; AP, anterior‐posterior; SI, superior‐inferior.

**Figure 2:**
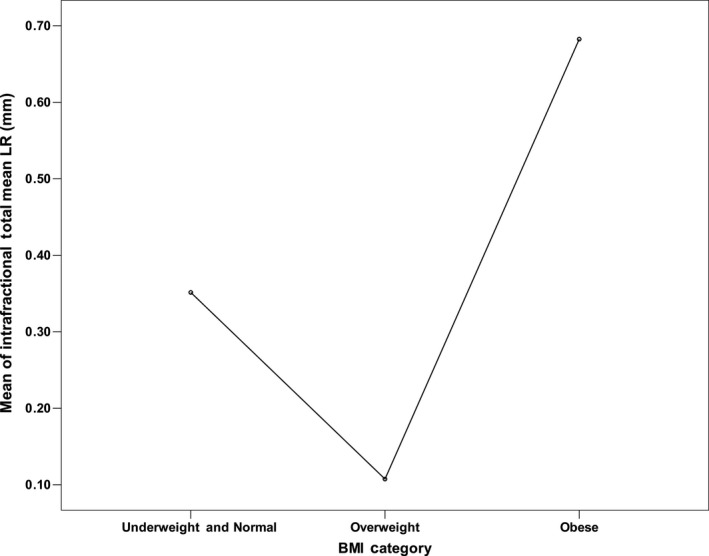
Intrafractional total mean left‐right (LR) motion across body mass index (BMI) categories.

Further investigation into each of the BMI categories was conducted. A one‐way ANOVA was conducted to determine if the mean prostate intrafraction motion differed across the BMI categories. The mean and standard deviations used in the ANOVA analysis are presented in Table 2. There was no statistical significance (*P* > 0.05) between any of the translations/rotations across BMI categories except LR (*P* = 0.003), as summarised in Table 4. Tukey HSD post hoc analysis indicated that the LR difference from overweight to obese (0.575, 95% CI (0.185–0.965)) was statistically significant (*P* = 0.002). The plot for the LR intrafractional mean motion is shown in Figure 2.

**Table 4:** One way ANOVA for intrafractional translations rotation

	ANOVA
LR (mm)	*F*(2, 130) = 6.126, *P* = **0.003**
AP (mm)	*F*(2, 130) = 0.743, *P* = 0.478
SI (mm)	*F*(2, 130) = 2.045, *P* = 0.134
Pitch (deg)	*F*(2, 130) = 1.646, *P* = 0.197
Yaw (deg)	*F*(2, 130) = 0.487, *P* = 0.616

The bold indicates a significant result. LR, left‐right; AP, anterior‐posterior; SI, superior‐inferior.

**Figure 3:**
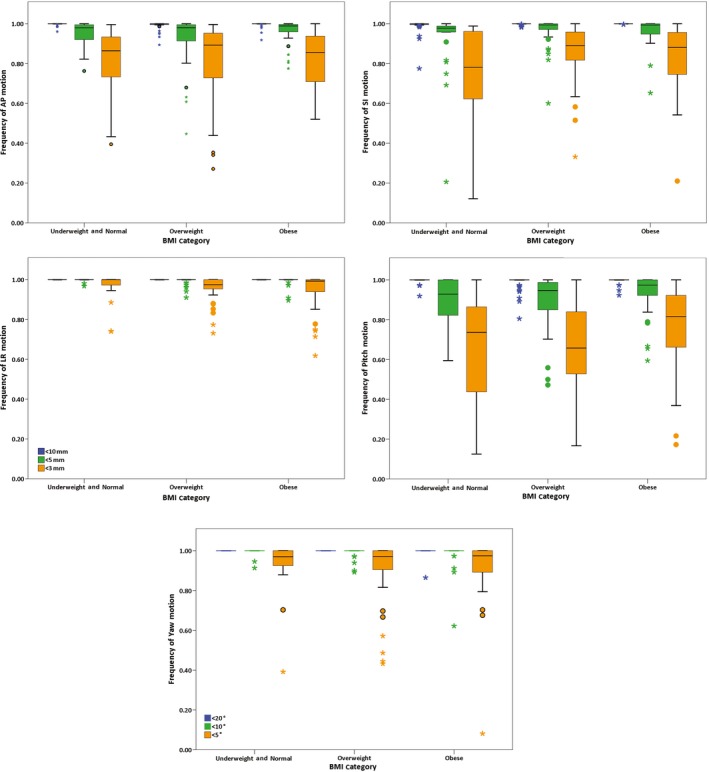
Boxplots of frequencies for intrafractional translations and measured rotations.

The frequency of the occurrence of displacement was investigated by recording the percentage displacements within 3 mm, 5 mm and 10 mm for translations, and 5, 10 and 20 degrees for rotations calculated by counting the number of frames within each given margin. The overall population results are summarised in Table 5, with BMI category results displayed in Figure 3.

**Table 5:** Frequency of translations and rotations within given margins, per fraction

	Overall population		
	Mean ± SD (%)	Min (%)	Max (%)
LR			
≤10 mm	100	100	100
≤5 mm	99.5 ± 2.0	99	100
≤3 mm	95.9 ± 7.0	62	100
AP			
≤10 mm	99.5 ± 2.0	89	100
≤5 mm	94.4 ± 9.0	45	100
≤3 mm	81.6 ± 16.0	27	100
SI			
≤10 mm	99.6 ± 2.0	78	100
≤5 mm	95.7 ± 1.0	21	100
≤3 mm	83.4 ± 17.0	12	100
Pitch			
≤20 deg	99.1 ± 2.6	81	100
≤10 deg	91.2 ± 11.7	47	100
≤5 deg	69.4 ± 22.4	13	100
Yaw			
≤20 deg	100	100	100
≤10 deg	99 ± 4.1	62	100
≤5 deg	91.8 ± 14.7	39	100

LR, left‐right; AP, anterior‐posterior; SI, superior‐inferior.

## Discussion

### Intrafraction motion

This study did not find any significant relationship between patient BMI and intrafractional prostate motion except in LR (*P* = 0.049), where the mean LR motion was 0.683 ± 0.856 mm for obese patients, compared to 0.352 ± 0.790 and 0.108 ± 0.760 mm for underweight/normal and overweight patients respectively.

The results indicated translational means and standard deviations were within current planning target volume (PTV) margins of 7–10 mm. While the mean intrafractional motion is an important indicator of motion, more extreme motions within a fraction, such as sudden transient motion may not be reflected in the mean or visualised in the movie captures. To investigate cases of more extreme motion, both the range of motion, and the frequency of displacement within 3, 5 and 10 mm were analysed. Current departmental margins from clinical target volume to PTV are 10 mm in LR, SI and anterior directions, and 5–7 mm in the posterior direction. Previous studies reducing the margins to as little as 3 mm have been performed.^11^, ^12^ When examining the overall population in this study, the percentage of the intrafractional motion within 3 mm was 83.4 ± 17.0 and 81.6 ± 16.0 for the SI and AP translations respectively. This agrees closely with the findings of Nichol et al., where motions of greater than 3 mm were observed in 12% of the total measurements (every 9 sec over 9 min total time on MRI scans).^13^

Two studies of note have investigated the correlation between BMI and intrafraction prostate motion. Using electromagnetic transponders, Butler et al., investigated the effect of BMI on prostate displacement. Their overall results based on 66 patients (mean BMI = 28.7 ± 4.2 kg/m^2^) show similar results.^14^ It should be noted that the patients were treated in a prone position with a custom thermoplastic hip‐fix immobiliser over the buttocks and abdomen. In separating the overall study population in to low BMI (<30 kg/m^2^) and high BMI (≥30 kg/m^2^) groups, no significant difference in the standard deviation of the translational motions or vector was found.^14^

Our standard deviation findings are supported by those of Thompson et al. Their conclusion was that while there was no statistically significant difference in the intrafraction between BMI categories, there may be greater stability of the prostate in the larger BMI patients, once isocentre correction has been made.^15^ Indeed, the standard deviations of patients >35 kg/m^2^ were within 2 mm in all translational directions, however, it should also be noted that there were only 8 patients in this sub‐group. Comparatively, our study had 21 patients with BMIs >35 kg/m^2^ and we did not find this trend, except for the LR direction.

### Rotation

A standard deviation of 3.57 degrees for pitch reflects a greater range in this rotation, a key finding of this study. Rotation remains a challenge in the IGRT setting, particularly in pitch which is influenced largely by rectal volume differences. Bowel protocols minimise these rectal differences, however, even if the patient has an acceptable rectal diameter on planning scan, a small difference in rectal diameter at treatment can produce a large difference in prostate displacement and rotation. This was further investigated by Oates et al., concluding that a mean rectal diameter measured on daily CBCT of less than or equal to 3.5 cm would result in a prostate displacement of less than or equal to 5.5 mm.^16^

Rotation was evident in our results, particularly when considering the magnitude and frequency of pitch (Table 5 and Fig. 3). While IGRT strategies allow for ease of translational corrections, there are currently limited options for the correction of rotational displacements. Current treatment couch tops with 6 degrees of freedom capabilities allow for the correction of small rotations, but not able to correct for larger rotations.

Pitch rotation is of particular concern if the seminal vesicles (SVs) are within the PTV. As the prostate is approximately spherical in shape, when treating prostate only, rotational error has limited possibility of underdosing the PTV. However, the addition of SVs within the PTV creates a much more irregularly shaped PTV, increasing the possible clinical significance of rotational errors. As 82% of this patient cohort included SVs for part or all of their treatment course, this is an important consideration. It was anecdotally noted that differences in rectal filling at the prostate level affected rotation and displacement. However, quantifying the rectal distension and/or shape changes was beyond the scope of this project.

### Limitations

A major limitation of this study is that the movie capture methodology does not provide real‐time 3D data, with only one aspect of the lateral or anterior treatment field collected and measured at any given time. Thus, movement in only two planes can be assessed for each movie capture series, with the inability to reliably capture large transient moves potentially occurring between fields or frames. These transient moves are measured within the literature, with Noel et al. detailing the risk of missing motion with intermittent imaging techniques.^17^, ^18^

Another limitation is the inability to measure prostate deformation from the fiducial markers on the movie captures. As such, the ‘best‐fit’ match introduces subjectivity, which was minimised through the measurement of intrafraction motion by one investigator. At present, there is no efficient method to measure prostate deformation and this was therefore beyond the scope of this project.

A small number of patients (*n* = 16, 12.3%) had only 1–2 fiducial markers present at time of treatment. The rate of loss of markers was higher than expected as this study was performed in conjunction with the implementation of fiducial marker insertion within the department, and thus represents the ‘learning curve’ for the procedure. Of these 16 patients, the majority (*n* = 11, 68.8%) were within the first 40 patients recruited. In these cases, the potentially less accurate measurement of motion, particularly in rotations is recognised.^19^

The effect of bowel and bladder filling is well‐known as a contributory factor to intrafractional motion.^20^, ^21^ Bladder and bowel filling protocols were adhered to in this cohort, and if bladder and/or bowel was significantly different on the pre‐treatment CBCT, corrective action would be undertaken by the treatment staff at their discretion. The bladder volumes were not investigated in this project, however, could be retrospectively further analysed based on the pre‐treatment CBCTs. Rectal volumes have been analysed on CBCT, and the effect on prostate intrafraction motion described by Oates et al.^16^

### Controversies in using BMI

The use of BMI as an indicator of obesity is contentious within the health community, despite its wide use. One of the main arguments against is the fact that a change in weight, and therefore BMI, does not necessarily reflect a change in obesity – particularly when exercise is proven to increase skeletal muscle mass.^22^ Alternative measures include waist circumference, hip‐to‐waist ratio, skin folds or body fat composition,^23^, ^24^ however, these may require additional measurements or specialised equipment. The advantage of BMI is that it is easily calculated from two measurements, height and weight, standardly measured in many health settings. This study included a validation of the relationship of BMI and pelvic adiposity by measuring pelvic adiposity on the planning CT (not reported in this paper).^25^

## Conclusion

Our findings did not support a relationship between intrafractional motion and BMI. The linear regression analysis did not find any statistically significant relationship and thus BMI could not predict the intrafractional motion. Our findings do support the ever‐growing body of evidence that highlights the importance of daily IGRT for the treatment of prostate cancer, and the progression towards intrafractional monitoring and correction. This is of particular importance in the advancements of EBRT treatments, including hypo‐fractionated schedules.

Our rotational results indicate the importance of correction for significant rotation, particularly pitch. The higher standard deviation in pitch when compared with the other rotations across the overall population is of note. This remains a great challenge in the EBRT for prostate cancer. These findings highlight the necessity for adherence to bladder and bowel protocol, and for the development of guidelines to inform the treatment therapists to take decisive action should there be considerable bladder/rectum volume changes on a daily basis.

In the continuing improvement of radiation therapy for prostate cancer, IGRT must continue to play an integral part. Advancements in the monitoring and correction of intrafractional motion will allow for safer dose escalation with the potential for reduced side effects, and improved quality of life.

## Conflict of Interest

The authors declare no conflict of interest.
